# The electronic properties of boron-doped germanium nanocrystals films

**DOI:** 10.1186/s11671-023-03893-7

**Published:** 2023-09-07

**Authors:** Dan Shan, Menglong Wang, Daoyuan Sun, Yunqing Cao

**Affiliations:** 1 School of Information Engineering, Yangzhou Polytechnic Institute, Yangzhou, 225127 China; 2 School of Physical Science and Technology/Microelectronics Industry Research Institute, Yangzhou University, Yangzhou, 225009 China

**Keywords:** Germanium nanocrystal, Electronic property, Temperature dependence Hall effect measurement, Scattering mechanism

## Abstract

Various doping concentrations of boron (B)-doped germanium nanocrystal (Ge NC) films were prepared using the plasma-enhanced chemical vapor deposition (PECVD) technique followed by thermal annealing treatment. The electronic properties of B-doped Ge NCs films combined with the microstructural characterization were investigated. It is worthwhile mentioning that the Hall mobilities ${\mu }_{\mathrm{Hall}}$ of Ge NCs films were enhanced after B doping and reached to the maximum of 200 cm^2^ V^−1^, which could be ascribed to the reduction in surface defects states in the B-doped films. It is also important to highlight that the temperature-dependent mobilities ${\mu }_{\mathrm{H}}(T)$ exhibited different temperature dependence trends in the Ge NCs films before and after B doping. A comprehensive investigation was conducted to examine the distinct carrier transport properties in B-doped Ge NC films, and a detailed discussion was presented, focusing on the scattering mechanisms involved in the transport process.

## Introduction

Over the past decade, there has been a growing interest in semiconductor nanocrystals based on Silicon and Germanium. These nanocrystals have gained attention due to their versatile applications in various devices, including light emitters, thin-film solar cells, photoelectric sensors, and nonvolatile memories [1–7]. Germanium, in particular, exhibits advantages such as larger electron and hole mobility, a narrower band-gap (0.67 eV), and high phonon responsivity in the near-infrared region [8–10]. These properties make it suitable for the fabrication of Ge-based photodetectors and Ge-based thin film transistors (TFT) with superior device performance [7, 11–13]. However, achieving desired properties in Si and Ge NCs often necessitates active doping to enhance device performance [14–17]. Unfortunately, doping nanocrystals is challenging, and the process is further complicated by the phenomenon known as "self-purification". This phenomenon makes it difficult for defects or impurities to migrate toward the surface of a nanocrystal due to the small distances involved [18, 19]. Consequently, there has been limited research conducted on the electronic properties of doped Si or Ge NCs, highlighting the need for a deeper understanding of the fundamental carrier transport properties in these materials [20–24].

In our previous works, the electronic properties of P- and B-doped Si NCs films were investigated by the temperature-dependent Hall effect measurements [20–22]. Compared with B dopants, P impurities are more likely to substitutionally occupy the inner sites Si NCs. They can be easily electrically activated and provide a large number of charge carriers, which finally reduces the barriers height of grain boundaries (GBs) in the Si NCs films. Thus, it was reported that the scattering effect of grain boundaries could be suppressed by the introduction of P impurities in Si NCs films. In the present work, the microstructure and carrier transport properties of B-doped Ge NCs films have been investigated. It was found that the crystallization of Ge NCs films was degenerated when B impurities were introduced. Almost no significant increases of carrier concentrations were observed in Ge NCs films after B doping, indicating the doping efficiency of B is low in Ge NCs materials. Furthermore, carrier transport processes in Ge NCs films before and after B doping were systematically studied. There were different scattering mechanisms dominated the carrier transport process, respectively, in Ge NCs films with various doping concentrations. The possible microscopic mechanisms which to govern the charge transport were briefly discussed.

## Experiment

B-doped hydrogenated amorphous germanium (*a*-Ge:H) films were prepared by a plasma-enhanced chemical vapor deposition (PECVD) system using the gas mixtures of pure germane (GeH_4_, Nanjing, China), hydrogen (H_2_, Nanjing, China) and diborane (B_2_H_6_, 1% diluted in H_2_, Nanjing, China). The flow rate of GeH_4_ was kept at 5 sccm (standard cubic centimeter per minute) during the growth process [20–22]. The boron concentrations were changed by adjusting the flow rate of B_2_H_6_ (*F*_B_), which controlled at 0 sccm (un-doped), 0.5, 1 and 3 sccm, respectively. The gas-chamber pressure, substrate temperature and radio frequency power were 10 m Torr, 250 ℃ and 30 W, respectively. The thickness of all samples is designed to be 150 nm. After deposition, all the samples were subsequently annealed in a conventional furnace at temperatures of 500 ℃ for 1 h in nitrogen ambient for crystallization. Quartz plates and monocrystalline Si wafers were selected as substrates for the various measurements [20–22].

The microstructures of all films were characterized using Raman spectroscopy (HR800, Jobin Yvon Horiba Inc.) and X-ray diffraction (MXP-III, Bruker Inc.). The optical band-gap of the films was determined by analyzing the optical absorption spectra obtained from a Shimadzu UV-3600 spectrophotometer using Tauc plots. The electronic properties, including dark conductivity, Hall mobility, and carrier concentration, were determined through temperature-dependent Hall measurements using a variable temperature Hall effect measurement system (model: LakeShore 8400 series, LakeShore). The measurements were conducted in a standard van der Pauw (VDP) configuration with an electromagnet generating a magnetic induction of 0.6 T. The temperature range for the Hall measurements was from 300 to 650 K. To achieve ohmic contacts, all samples were prepared with coplanar Al electrodes at the four corners using vacuum thermal evaporation, followed by a 30-min alloying treatment at 400 °C.

## Results and discussion

### Microstructure characterization

**Fig. 1:**
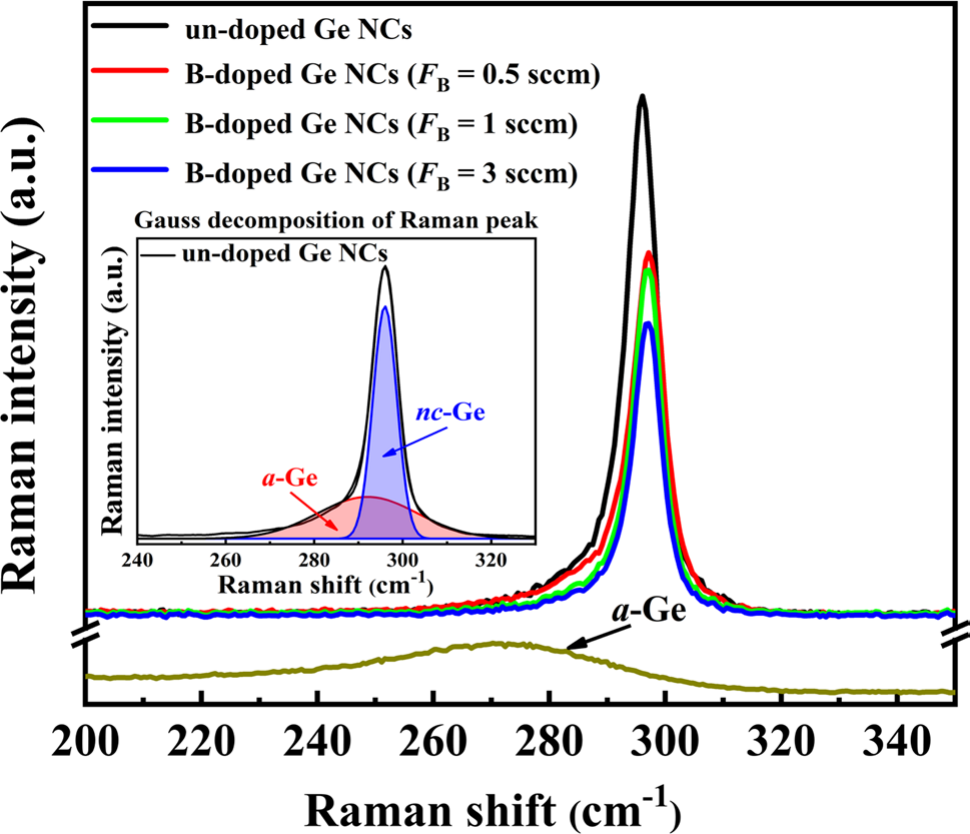
the Raman spectrum of Ge NCs films with various B doping concentrations. The *inset* is the Gauss decomposition of Raman peak

Raman spectroscopy, commonly employed to investigate the microstructures of annealed nanocrystalline films, was conducted using an Ar + laser with a wavelength of 514 nm as the excitation light source. Figure 1 displays the Raman spectra of B-doped Ge NC films with different doping concentrations. A weak and broad Raman band centered at 273 cm^−1^ is observed in the as-deposited film, which is indicative of the transverse-optical (TO) vibration mode of amorphous Ge–Ge bonds. Conversely, in the samples subjected to the annealing procedure, a sharp peak emerges near 300 cm^−1^, indicating the formation of Ge NCs phases [23, 25]. The volume fraction of crystalline ${X}_{\mathrm{C}}$ can be estimated as the formula: ${X}_{\mathrm{C}}=\frac{{I}_{\mathrm{c}}}{{I}_{\mathrm{c}}+\sigma {I}_{\mathrm{a}}}$ [26]. To analyze the Raman spectra, a deconvolution process was employed, separating the spectrum into two Gaussian components representing nanocrystalline (*nc*-Ge) and amorphous (*a*-Ge) phases. The deconvolution results are depicted in the inset of Fig. 1. The integrated intensities of the crystalline and amorphous peaks are represented by ${I}_{\mathrm{c}}$ and ${I}_{\mathrm{a}}$, respectively. In our analysis, a value of *σ* equal to 0.88 was used, signifying the ratio of the integrated Raman cross sections between the crystalline and amorphous phases. It can be calculated that ${X}_{\mathrm{C}}$ is about 86% for the un-doped Ge NCs film and was gradually decreased to nearly 65% for B-doped Ge NCs film with the maximum doping amount of *F*_B_ = 3 sccm. Similar results in Si NCs films had been reported at our preliminary work [27]. The reduction in ${X}_{\mathrm{C}}$ was primarily attributed to the introduction of dopants, resulting in increased fluctuations in bond angles and bond lengths as the dopants were incorporated into the films. This phenomenon led to the degradation of the short-range order. From the aforementioned discussion, we can conclude that the incorporation of B dopants results in reduced crystallization within the Ge NCs films.

**Fig. 2:**
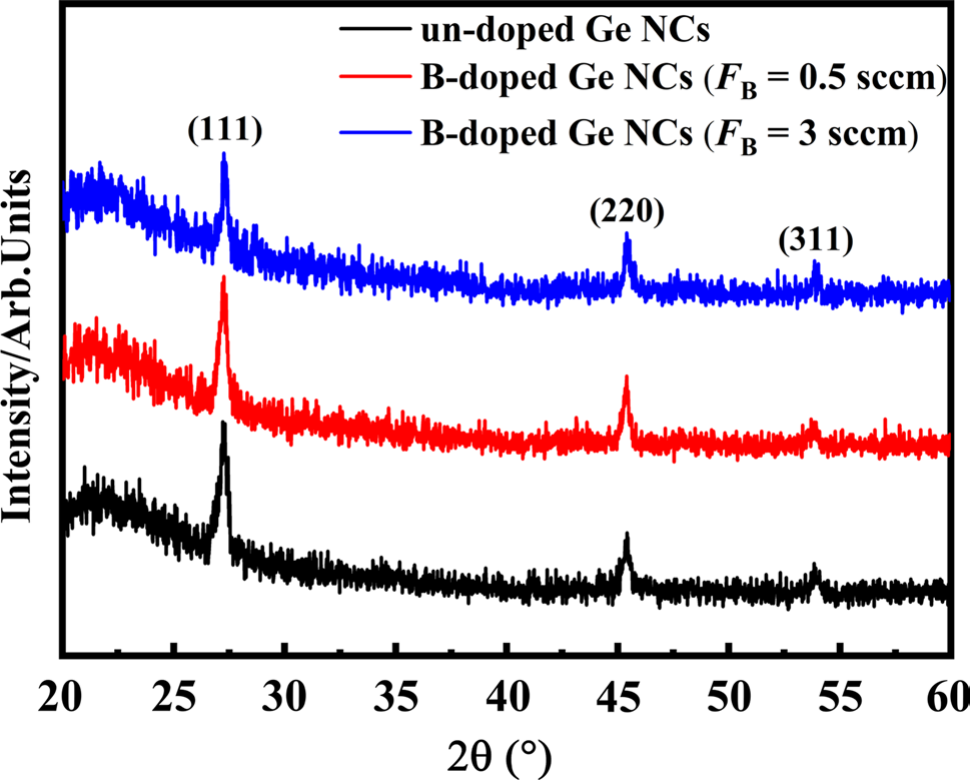
The XRD patterns of Ge NCs films with various B doping concentrations

The microstructure of B-doped Ge NCs films with various doping concentrations was further investigated by XRD measurement. The XRD patterns of all samples deposited on quartz substrates are shown in Fig. 2. It was clearly observed that the diffraction peak at near 2$\theta$=27° and 45°, which associated with the poly-crystalline planes of (111) and (220) for Ge, respectively, appeared in all the films [28]. Moreover, the diffraction peak corresponding to the (111) plane in Ge NCs films exhibits a slight reduction in intensity following B doping. This suggests that the proportion of crystalline material in the films may have decreased as a result of B doping [29]. This observation implies that the introduction of B dopants can adversely affect the quality of Ge NCs, leading to a decrease in crystallization within the Ge NCs films. These findings are consistent with the results obtained from the Raman analysis.

**Fig. 3:**
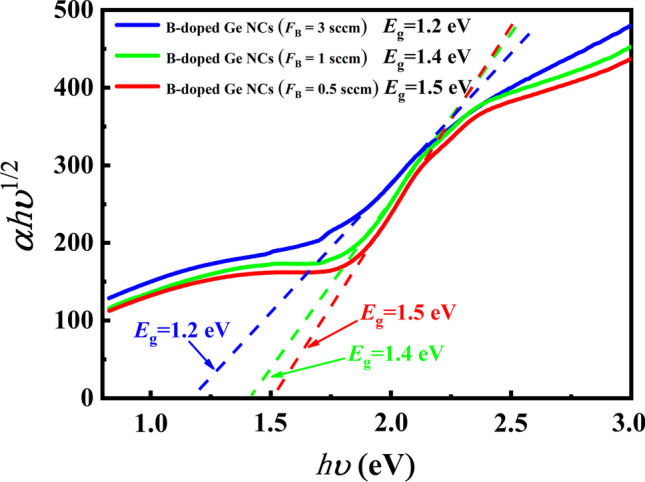
the Tauc’s plot of ${(\alpha h\nu )}^{1/2}$ versus photon energy $h\nu$ for Ge NCs films with various B doping concentrations

The optical band-gap *E*_g_ is commonly determined using Tauc plots, where ${(\alpha h\nu )}^{1/2}=B(h\nu -{E}_{\mathrm{g}})$. Here, $\alpha$ represents the absorption coefficient, $h\nu$ is the photon energy, and *B* is a constant. This method is employed to characterize the light absorption in nanocrystalline semiconductor films [30, 31]. Figure 3 shows the Tauc’s plot of ${(\alpha h\nu )}^{1/2}$ versus photon energy $h\nu$ for B-doped Ge NCs films. It is found that the value of optical band-gap, which was reported as 1.6 eV for the un-doped Ge NCs film in our previous work [32], was slightly reduced to 1.5 eV for the B-doped Ge NCs with *F*_B_ = 0.5 sccm and dropped to 1.2 eV when the B doping concentration increased to *F*_B_ = 3 sccm. The optical band-gap is known to be influenced by the presence of disordered grain boundary regions in the crystallized sample, which typically exhibit a higher optical band-gap compared to the nanocrystalline regions [33]. The widespread distribution of grain boundary regions in Ge NC films contributes significantly to the overall optical band-gap. Therefore, the decrease in optical band-gap for the B-doped Ge NCs films may be ascribed to the decline of nanocrystalline components caused by the introduction of B dopants that have already been described in the above.

### Room temperature Hall effect measurement

**Fig. 4:**
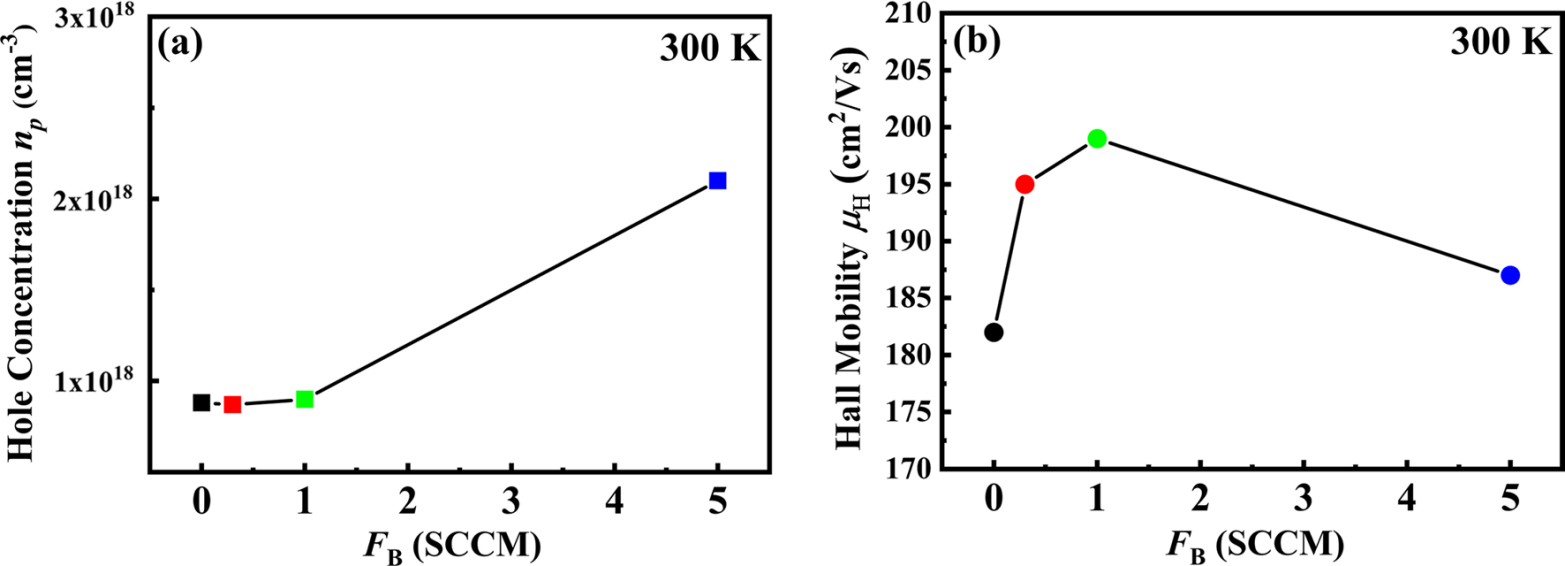
**a** Room temperature carrier concentrations and **b** Room temperature Hall mobilities for Ge NCs films before and after B doping

In order to gain a deeper understanding of the electronic properties in B-doped Ge NCs films at different doping concentrations, Hall effect measurements were employed, which was utilized to provide enhanced insights into the behavior of carriers in the Ge NCs films. In Fig. 4a, room temperature carrier concentrations for Ge NCs films before and after B doping are presented. It was interested to find that the carrier concentration of un-doped Ge NCs film was nearly reached to 10^18^ cm^−3^ order with a p-type behavior. It was reported that the presence of a significant hole concentration in Ge NCs film, even without deliberate doping, can be attributed to the existence of deep-acceptor-like surface states. These surface states, often associated with dangling bonds (DBs), result in the accumulation of numerous negative charges at the surfaces [32]. As a result, the energy bands in the vicinity of the surface exhibit an upward bending, thereby attracting additional holes within the film. Consequently, the un-doped Ge NCs film experiences a pronounced increase in hole concentration. However, the hole concentration was barely increased in Ge NCs films after B doping as if the dopants could not enter into the Ge NCs. As we know, the dopants may have difficulty entering the core of nanocrystalline Si or Ge according to the so-called self-purification effect, especially the Si or Ge NCs with small sizes [18, 34]. Therefore, there is no more activated impurities that can contribute to the carrier concentration in the B-doped Ge NCs films.

Furthermore, the Hall mobilities ${\mu }_{\mathrm{Hall}}$ in B-doped Ge NCs films were also investigated at room temperature as shown in Fig. 4b. The Hall mobility is about 182 cm^2^ V^−1^ in Ge NCs film before doping, exhibiting a normal electrical performance compared with other reports [32]. After doping, it is very interesting to find that the value of ${\mu }_{\mathrm{Hall}}$ is higher than that of the un-doped sample. The Hall mobility was firstly enhanced and reached to nearly 200 cm^2^ V^−1^ for B-doped Ge NCs film with *F*_B_ = 1 sccm, then decreased with increasing the B doping concentration. As we are aware, the carrier mobility in bulk Si is typically reduced as the doping concentration increases, primarily due to the strong scattering effect caused by impurities. However, the situation is quite different in the case of B-doped Ge NCs, where we observe an enhanced Hall mobility. This unconventional behavior can be attributed to several factors which will be examined in detail.

The relatively low carrier mobility observed in poly-crystalline and nanocrystalline Si or Ge is typically attributed to two primary factors: strong scattering at grain boundaries and the presence of defect states, which encompass the crystalline quality and interface defect states. Notably, the dangling bonds play a significant role in this regard, as they have the ability to trap carriers and generate a depletion region that subsequently affects carrier mobility [35–37]. However, the surface defects states can be well-passivated by the dopants. In our previous research, we investigated the effects of phosphorus (P) doping in Si NCs and made a significant discovery. We found that the introduction of P impurities effectively passivated the dangling bonds within the Si NCs. This passivation process played a crucial role in enhancing the electron mobility within the P-doped Si NCs, as we documented in our previous study [21]. In contrast to phosphorus impurities, boron impurities exhibit a distinct preference for occupying surface sites on nanocrystals rather than settling within the core of the nanocrystals. Hong et al. made an additional observation regarding the behavior of B atoms in Si NC films. They found that B atoms initially substituted the inactive threefold Si atoms present in the defect states of the Si NC films. Subsequently, these B atoms replaced the fourfold Si atoms, leading to electrically active doping [38]. Based on the preceding discussion, it becomes evident that B dopants, which occupy the surface sites, effectively passivate the defect states. Therefore, the observed enhancement in Hall mobility following B doping can be attributed to the reduction in surface defect states in our current study.

### Temperature-dependent Hall effect measurement

**Fig. 5:**
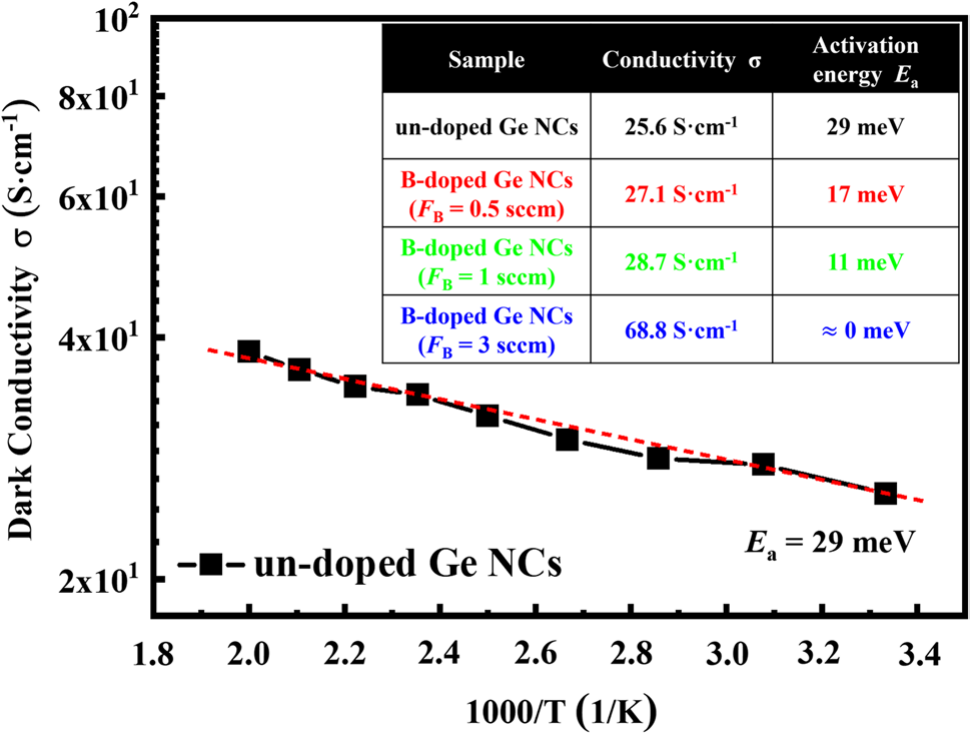
temperature-dependent dark conductivity for un-doped Ge NCs film; the insert are the deduced activation energy ${E}_{a}$ for Ge NCs films with various B doping concentrations

To gain a deeper understanding of the transport properties of B-doped Ge NC films, particularly regarding the scattering mechanisms involved in the transport process, we conducted an investigation of temperature-dependent Hall effects, which covered a temperature range spanning from 300 to 650 K. As shown in Fig. 5, temperature-dependent dark conductivity for un-doped Ge NCs film was measured at the temperature from 300 to 500 K. It was clearly found that the sample exhibited a linear relationship of the $\mathrm{ln}\sigma$ versus ${T}^{-1}$ plot, which indicates that carrier transport within the Ge NCs film is primarily governed by thermal activation conduction [29]. The activation energy, ${E}_{\mathrm{a}}$, representing the energy difference between the Fermi level and the top of the valence band in p-type semiconductors, can be determined by analyzing the slope of $\mathrm{ln}\sigma$ versus ${T}^{-1}$ curve according to the Arrhenius plots $\sigma ={\sigma }_{0 }\mathrm{exp}$($-{E}_{\mathrm{a}}/{k}_{\mathrm{B}}T$). The deduced activation energy ${E}_{\mathrm{a}}$ of the un-doped Ge NCs films is only about 29 meV and gradually decreased to nearly 0 meV after B doping with a series of doping concentration (insert of Fig. 5). Compared with the band-gap of 1.6 eV for the un-doped Ge NCs film, the relatively small activation energies observed in all the Ge NC films can be attributed primarily to the presence of deep-acceptor-like surface states. As mentioned earlier, these surface states lead to the accumulation of holes and cause the Fermi level to be pinned near the top of the valence band.

**Fig. 6:**
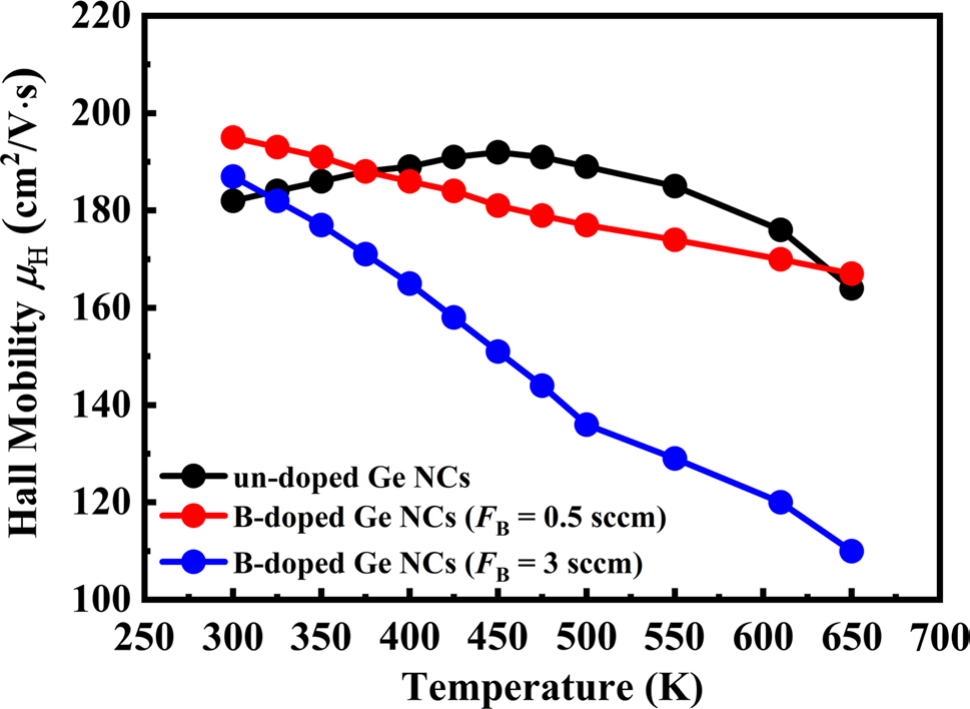
The temperature dependence Hall mobilities of Ge NCs films with various B doping concentrations

To extract information about the scattering mechanisms in the transport process, temperature-dependent Hall mobilities were investigated at the temperature range from 300 to 650 K. As can be seen from Fig. 6, it should be pointed out that the curves of temperature dependence Hall mobilities have different trends for Ge NCs films before and after B doping, which implies different scattering mechanisms in these samples. For the un-doped Ge NCs film, the mobility is firstly increased with increasing the temperature to near about 450 K and then, decreased with further increasing the temperature. Considerable research efforts have been devoted to the increased Hall mobility with temperature in the previous works including our reports [22, 39, 40]. It can be interpreted that the carrier transport process is mainly dominated by the grain boundaries scattering in un-doped Ge NCs film at room temperature. The grain boundaries, characterized by a higher concentration of defects, tend to trap charged carriers to a greater extent than the interior of the grains. As a result, band bending occurs at the grain boundaries, impeding carrier transport. However, as the temperature increases, the carriers gain additional energy, facilitating easier traversal of the potential barriers at the grain boundaries. Consequently, the mobility exhibits an increase with temperature, attributed to the reduced scattering caused by the grain boundaries [39].

**Fig. 7:**
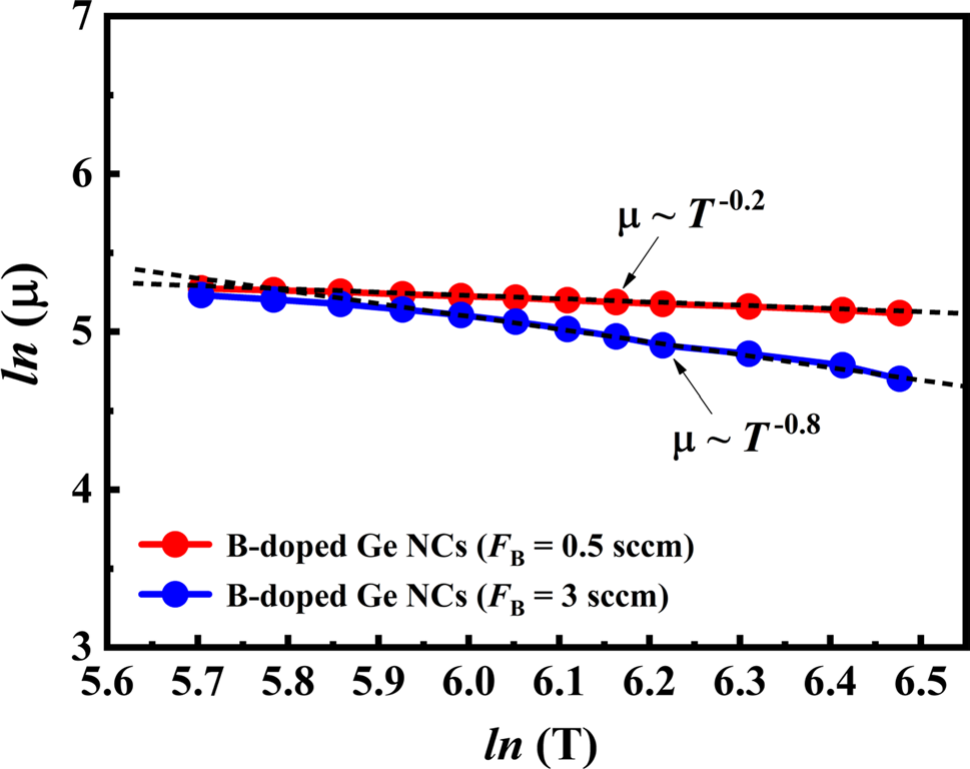
The $\mathrm{log}{\mu }_{H}$ as a function of $\mathrm{log}T$ for the B-doped Ge NCs film with *F*_B_ = 0.5 and 3 sccm in the temperature range from 300 to 650 K

However, the mobilities exhibited different temperature dependence trends after B doping, especially in the temperature region 300–450 K. It can be found that the Hall mobility decreases monotonously with the temperature in the whole measurement temperature range, which implies that the grain boundaries scattering may not dominate the carrier transport process in B-doped Ge NCs films. We suggested it can be ascribed to the reduction in crystallization in Ge NCs films after B doping. The lower crystallization in Ge NCs films, the fewer and smaller grains spreading over the films that due to the decrease in grain boundaries. Thus, the grain boundaries scattering mechanism could not play an important role in the carrier transport process in B-doped Ge NCs films. To further explore the scattering mechanism in B-doped Ge NCs films, Fig. 7 plots the $\mathrm{log}{\mu }_{H}$ as a function of $\mathrm{log}T$ in the temperature range from 300 to 650 K. The ${\mu }_{\mathrm{H}}(T)$ can be described by the following equation ${\mu }_{\mathrm{H}}(T)$ $\propto$ ${T}^{n}$. It can be estimated that the data yield *n* = − 0.8 for the B-doped Ge NCs film with *F*_B_ = 0.5 sccm and *n* = − 0.2 for the B-doped Ge NCs film with *F*_B_ = 3 sccm. It is generally believed that typical scattering is acoustic phonons, ionized and neutral impurities scattering, which yield values of − 1.5, 1.5 and 0, respectively [41]. However, in our present works, the B dopants are more likely to occupy the surface sites of Ge NCs dots than be ionized to realize electrically active doping. Therefore, the exponents, which show the *n*-value of about − 0.2 and − 0.8, indicate that the carrier transport is dominated by a superposition of acoustic phonon scattering, neutral impurities scattering as well as grain boundaries scattering [42]. It must also be mentioned that the grain boundaries scattering is weaker in the B-doped Ge NCs film with *F*_B_ = 3 sccm than in the film with *F*_B_ = 0.5 sccm. Consequently, the *n*-value of − 0.8 for the B-doped Ge NCs film with *F*_B_ = 3 is shown closer to − 1.5, which indicates the acoustic phonon scattering should play a more and more important role in the Ge NCs films after B doping.

## Conclusion

In summary, B-doped Ge NCs films with various doping concentrations were fabricated by thermal annealing of the corresponding *a*-Ge:H films. Both the crystallinity and average grain size in Ge NCs films were gradually decreased with increasing B doping concentrations. A high hole concentration could be achieved to more than 10^18^ cm^−3^ order due to the holes accumulation caused by the acceptor-like surface states in Ge NCs film. However, the hole concentrations in Ge NCs films after B doping had barely increased, which implies that the B atoms hardly enter into the core of Ge NCs to realize electrically active doping. Another interesting finding is that the Hall mobilities in Ge NCs films were unusually increased after B doping, which can be ascribed to the reduction in surface defects states well-passivated by the dopants. Based on the temperature-dependent Hall effect measurement, the scattering mechanisms during carrier transport process in Ge NCs films were investigated. It can be found that different scattering mechanisms were observed in the Ge NCs films before and after B doping. In un-doped Ge NCs film, the grain boundaries scattering mainly dominated the carrier transport process. After B doping, the carrier transport process was dominated by a superposition of acoustic phonon scattering, neutral impurities scattering and grain boundaries scattering and the acoustic phonon scattering played a more and more important role when further increasing the B doping concentration.

## Data Availability

The data used to support the findings of this study are available from the corresponding authors upon request.

## Abbreviations

Ge NCs: Germanium nanocrystals
PECVD: Plasma-enhanced chemical vapor deposition
a-Ge:H: Hydrogenated amorphous germanium
SCCM: Standard cubic centimeter per minute
XRD: X-ray diffraction
GBs: Grain boundaries
DBs: Dangling bonds
